# In Silico Analysis of Bioactive Compounds from Leaves of Abrus precatorius L. (Rosary Pea) Against Breast Cancer

**DOI:** 10.7759/cureus.67808

**Published:** 2024-08-26

**Authors:** Harishkumar Baskaran, Thirumal Margesan, Kamaraj Raju

**Affiliations:** 1 Pharmacognosy, SRM (Sri Ramasamy Memorial) College of Pharmacy, SRM Institute of Science and Technology, Kattankulathur, IND; 2 Pharmacy, SRM (Sri Ramasamy Memorial) College of Pharmacy, SRM Institute of Science and Technology, Kattankulathur, IND

**Keywords:** her 2 receptors, progesterone receptor, molecular docking, 4-dimethyl-4 pentenyl acetate, osiris, pass software, molinspiration, breast cancer, abrus precatorius

## Abstract

Introduction

Breast cancer is one of the most common causes of cancer among women. Since the administration of chemotherapy drugs can cause several adverse effects, thus it leads to research on effective treatment from natural sources. Leaves of *Abrus precatorius *L., a member of the *Fabaceae* family,contain several medicinal properties. It has drawn interest as an anti-cancer agent since its leaves contain different phytochemicals that can cause apoptosis in a variety of cancer types.

Methods

A total of 97 compounds were identified from the ethyl acetate extract of *A. precatorius* leaves by gas chromatography/mass spectrometry (GC/MS) analysis. Of those, eight compounds were selected based on the percentage area above 2. Cheminformatics software such as Molinspiration (Molinspiration Cheminformatics free web services, Slovensky Grob, Slovakia) was used to predict the molecular properties and bioactivity. PASS software (NCSS LLC, Kaysville, Utah, United States) was used to predict the scores of anticancer properties such as antioxidant, anti-inflammatory, immunosuppressant, antineoplastic, and cytoprotective. Osiris Property Explorer software was used to determine pharmacokinetic profile and toxicity prediction, and molecular docking was performed to determine the binding affinity towards the receptors.

Results

Out of eight compounds, one was selected based on the scores of the above software, then docking studies were done by using AutoDock Vina 4.2.6 (Center for Computational Structural Biology, La Jolla, California, United States). The results were compared with the reference compound, 5-fluorouracil, and 1,4-dimethyl-4 pentenyl acetate was identified as the most promising active compound found in this study. It shows better binding affinity towards the progesterone receptor (-6.0) when compared to the reference compound.

Conclusion

Based on the results, it has been proved that 1,4-dimethyl-4 pentenyl acetate may be used as an alternative for the management of breast cancer.

## Introduction

Breast cancer is an extremely prominent type of cancer and one of the leading causes of mortality. Globally, around 1.5 million women get diagnosed with breast carcinoma annually. If cancer is detected early, there is a high possibility of rehabilitation and survival [1]. Estrogen receptor (ER), human epidermal growth factor receptor 2 (HER2), and progesterone receptor expression status show significant heterogenicity in breast cancer and can be used to categorize the disease into distinct classes [2]. Aging is one of the primary warning signs for developing breast cancer. Other important risk factors include family history (BRCA1/2 mutations), reproductive variables (early menarche and late menopause), imbalances in hormones (increased estrogen levels), and lifestyle choices (high fat and alcohol intake).

About 5-10% of breast cancer cases are estimated to have a genetic basis; of these hereditary cases, BRCA1/2 genetic mutations account for approximately 50% of the occurrences [3]. According to the Global Cancer Network, an estimated 19.3 million incident cancer cases in 2020 and 10 million deaths are caused by cancer globally. Cancers of the breast (15.5%), lungs (13.7%), and cervix (7.7%) were the causes of death among women. With 287,850 incidents in 2022, breast carcinoma remains the extremely prevalent cancer in terms of the number of cases reported each year. In females, the leading incidence of cancer is breast cancer (30%), lung and bronchus cancer (13%), colorectal cancer (8%), uterine corpus (7%), and skin cancer (4%) and the majority of deaths are due to lung and bronchus cancer (23%), breast cancer (15%), and colorectal cancer (8%) [4].

Endocrine treatments are used to treat breast cancers that are ER-positive, which are usually started following surgery. These treatments include aromatase inhibitors like letrozole and ER down regulators and modulators like fulvestrant and tamoxifen, respectively. Treatment for HER2 malignancies includes HER2-targeting medications such as trastuzumab and lapatinib [2]. There is a need for more selective and efficient medications for the therapy of breast cancer. Triple-negative breast cancer, the subtype regarded to be more malignant, lacks selective treatment and is dependent on non-specific chemotherapeutics. Chemotherapy, on the other hand, is a general treatment with serious side effects that significantly lower patients' quality of life. Furthermore, chemotherapeutic medicines might cause drug resistance if they are exposed to patients for an extended period of time. Chemotherapy medications used in large doses for patients with drug-resistant malignancies often have few or no beneficial effects. The invention of novel chemotherapy drugs that may offer chronic cancer management with few complications can be largely attributed to natural products [3].

*Abrus precatorius* L., a member of the *Fabaceae* family, is a potentially useful herbal plant for natural medicine. Tropical and subtropical regions of the world are suitable for the growth of *A. precatorius* [5]. It is commonly known as rosary pea or jequirity bean in English and there are many Indian names such as Gunj, Gumchi, Chanoti, and Chirmiti. For many years, tribal communities have used the leaf of *A. precatorius* in their folk remedies. It is said to have a variety of therapeutic effects, including antifungal, antitumor, analgesic, anti-inflammatory, antispasmodic, antibacterial, anti-diabetic, and antiserotonergic properties [6]. *A. precatorius* has drawn interest as an anti-cancer agent because it has been demonstrated that different phytochemicals from the leaves can cause apoptosis in a variety of cancer types. Secondary metabolites isolated from *A. precatorius* include flavonoids, isoflavanoquinones, anthocyanins, tannins, steroids, alkaloids, phenolic compounds, proteins, and other triterpenoids [7]. Recent medical research has focused on plant-derived antioxidants, particularly polyphenols and flavonoids, because they are bioactive substances with anticancer, antidiabetic, antimicrobial, hepatoprotective, neuroprotective, and cardioprotective properties [8]. The fraction of ethyl acetate and butanol-modulated cytokines and antiproliferative activities indicate the potential use of chemotherapy in the treatment of cancer [9]. An ester derivative obtained from ethyl acetate extract of *A. precatorius *leaves, 2-linoleoylglycerol contains an IC50 value of 13.2 µg/ml, in MDA-MB-231 breast cancer cell lines [7].

In the present study, the compounds present in the ethyl acetate extract of *A. precatorius *leaves were analyzed through gas chromatography/mass spectrometry (GC/MS) and to reduce cost and time, instead ofin vitro and in vivo studies, computer-aided drug design (CADD) was used to identify the active drug candidate for anti-breast cancer therapy.

## Materials and methods

This was an in silico study conducted at the SRM (Sri Ramasamy Memorial) College of Pharmacy, SRM Institute of Science and Technology, Kattankulathur, Tamil Nadu, India.

Prediction of molecular properties and bioactivity

A total of 97 compounds obtained from GC/MS analysis were shortlisted by selecting the compounds that have a percentage area above 2, and of these, eight compounds were selected for further study (Table 1).

**Table 1:** GC/MS identification of compounds from ethyl acetate extract of Abrus precatorius GC: gas chromatography; MS: mass spectrometry

SI. No	Retention Time	Area	Area (%)	Height	Compound name
1.	15.065	94150	3.67	26607	1,1-difluoro dodecane
2.	15.361	102272	3.99	37547	3,3-bis(tert-butylsulfanyl)-2-fluoroacrylonitrile
3.	16.473	53613	2.09	19642	phthalic acid, 4-cyanophenyl nonyl ester
4.	17.567	72556	2.83	12425	1-iodo dodecane
5.	17.731	78061	3.05	27253	3,3-dimethyl hexane
6.	19.191	202730	7.91	73839	1-octadecyne
7.	19.659	112577	4.39	26000	1,4-dimethyl-4-pentenylacetate
8.	20.119	182115	7.11	60948	heptacosanoic acid, methyl ester

The molecular properties and bioactivity scores were predicted by utilizing Molinspiration software (Molinspiration Cheminformatics free web services, Slovensky Grob, Slovakia). The structures of the compounds selected were referred from the PubChem database, drawn on the Molinspiration software, and generated to predict the molecular properties and bioactivity scores [10].

Prediction of biological activity

The selected compounds were collected in canonical simplified molecular input line entry system (SMILES) notation. The probability of anticancer action was predicted using PASS software (NCSS LLC, Kaysville, Utah, United States). Inhibition of antioxidant, anti-inflammatory, immunosuppressant, antineoplastic, and cytoprotective activities was recorded based on their probability to be active (Pa) score [11].

Toxicity profile of compound

The compounds with a Pa score greater than 0.5 were analyzed for toxicity studies. The Osiris Property Explorer software was utilized for predicting toxic effects; the findings shown in red color indicate the toxicity, while the green color indicates the compound with drug-like properties [12].

Molecular docking studies

Preparation of Ligand

For molecular docking studies, 1,4-dimethyl-4-pentenylacetate was selected. PubChem, a free online server to study the structure and properties of chemical compounds, was used to obtain the three-dimensional (3D) structure of the compound, which was saved in .sdf formats [13]. The ligands were converted to Protein Data Bank (PDB) format using Open Babel software [14]. From the prepared ligands, the docking was carried out by utilizing AutoDock Vina 4.2.6 (Center for Computational Structural Biology, La Jolla, California, United States) [15].

Preparation of Protein Structure

The 3D structures of protein targets named were collected from the Research Collaboratory for Structural Bioinformatics (RCSB) PBD and saved in PDB format [16]. The BIOVIA Discovery Studio visualizer (Dassault Systèmes SE, Vélizy-Villacoublay, France) was used to prepare the protein by removing water molecules, hetatoms, ligands, and addition of lost hydrogen atoms placed on the protein structure. The prepared protein along with active sites were saved in .pdb format [17].

Molecular Docking Analysis

Autodock Vina 4.2.6 were used for docking of ligands onto the protein targets such as Erα (PDB ID 3ERT), ERβ (PDB ID 1QKM), HER 2 (PDB ID 3PP0), hBRD4 (PDB ID 4ZW1) and progesterone receptors (PDB ID 1E3K). The results were evaluated to identify the lowest binding energy and noted. The interaction between amino acids and the ligands was visualized by the BIOVIA Discovery Studio visualizer [18].

## Results

GC/MS identification of compounds from ethyl acetate extract of *A. precatorius*

**Figure 1:**
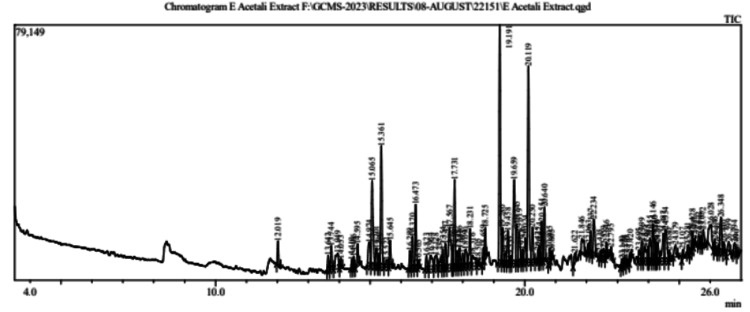
GC/MS chromatogram of ethyl acetate extract of Abrus precatorius leaves GC: gas chromatography; MS: mass spectrometry

The presence of important phytochemical substances including 1-octadecyne, heptacosanoic acid methyl ester, 1,1, difluoro dodecane, 1-iododecane, 3,3-dimethyl hexane, phthalic acid-4-cyanophenyl nonyl ester, 3,3 bis(tetr-butyl sulfanyl)-2-fluoroacrylonitrile and 1,4-dimethyl-4-pentenyl acetate is shown in Table 1 and the chromatogram is given as Figure 1.

Prediction of molecular properties

The compounds selected from the GC/MS identification of ethyl extract of *A. precatorius l*eaves were predicted for their molecular properties using Molinspiration software to identify the compounds that follow the Lipinski rule of 5 (Table 2).

**Table 2:** Predicted molecular properties of ethyl acetate extract of Abrus precatorius using the Molinspiration software* *Molinspiration Cheminformatics free web services, Slovensky Grob, Slovakia TPSA: topological polar surface area; MW: molecular weight; OH: oligonucleotides

Compound names	miLogP	TPSA	natoms	MW	nON	nOHNH	nviolations	nrotb	Volume
1-octadecyne	8.39	0.00	18	250.47	0	0	1	14	303.48
Heptacosanoic acid, methyl ester	9.78	26.30	30	424.75	2	0	1	26	493.77
Dodecane, 1,1-difluoro-	6.36	0.00	14	206.32	0	0	1	10	223.90
Decane, 1-iodo-	5.79	0.00	11	268.18	0	0	1	8	204.40
Hexane, 3,3-dimethyl-	3.79	0.00	8	114.23	0	0	0	3	145.79
3,3-bis(tert-butylsulfanyl)-2-fluoroacrylonitrile	3.78	23.79	15	247.4	1	0	0	4	230.47
Phthalic acid, 4-cyanophenyl nonyl ester	6.97	76.40	29	393.48	5	0	1	13	379.22
1,4-dimethyl-4-pentenylacetate	2.48	26.30	11	156.22	2	0	0	5	168.45
5 fluorouracil	-0.59	65.72	9	130.08	4	2	0	0	96.91
Letrozole	2.12	78.30	22	285.31	5	0	0	3	254.46

Prediction of bioactivity

The compounds showing zero violation follow the Lipinski rule for oral bioavailability. The predicted bioactivity scores of ethyl acetate extract of *A. precatorius* by Molinspiration software are shown in Table 3. 

**Table 3:** Predicted bioactivity scores of ethyl acetate extract of Abrus precatorius by Molinspiration software* GPCR: G protein-coupled receptors *Molinspiration Cheminformatics free web services, Slovensky Grob, Slovakia

Compound name	GPCR ligand	Ion channel modulator	Kinase inhibitor	Nuclear receptor ligand	Protease inhibitor	Enzyme inhibitor
1,4-Dimethyl-4 pentenyl acetate	-1.02	-0.40	-1.58	-0.60	-1.02	-0.22
3,3-Bis-tert-butylsulfanyl-2-fluoro-acrylonitrile	-0.45	-0.30	-0.95	-0.42	0.07	-0.03
3,3-Dimethylhexane	-2.98	-2.65	-3.65	-2.81	-3.12	-2.55
5 fluorouracil	-2.60	-1.95	-2.62	-3.04	-3.15	-1.56
Letrozole	-0.05	-0.08	-0.19	-0.25	-0.23	0.30

Prediction of biological activity

The pharmacological properties related to anticancer activity were predicted for the compounds selected from the Molinspiration software and checked for their Pa score, as shown in Table 4.

**Table 4:** Biological activity scores of ethyl acetate extract of Abrus precatorius were predicted using PASS software* *NCSS LLC, Kaysville, Utah, United States Pa: probability to be active; Pi: probability to be inactive

Compound name	Anti-oxidant		Anti-inflammatory		Immunosuppressant		Anti-neoplastic		Cytoprotective	
	Pa	Pi	Pa	Pi	Pa	Pi	Pa	Pi	Pa	Pi
1,4-Dimethyl-4-pententl acetate	0.296	0.024	0.733	0.012	0.604	0.023	0.638	0.029	0.412	0.092
3,3-Bis-tert-butylsulfanyl-2-fluoro-acrylonitrile	-	-	0.340	0.045	0.224	0.168	0.122	0.070	0.455	0.076
3,3-Dimethyl hexane	-	-	0.430	0.016	-	-	0.134	0.059	0.604	0.029
Letrozole	-	-	0.355	0.038	-	-	0.739	0.020	0.304	0.182
5-fluoro uracil	-	-	0.274	0.123	0.463	0.050	0.919	0.005	0.441	0.080

Molecular docking

In Osiris Property Explorer, 1,4-dimethyl-4-pententyl acetate is nontoxic for the parameters; hence, this compound is further subjected to molecular docking for the selected proteins, which was compared with the standard 5 fluorouracil. The binding affinities are shown in Table 5.

**Table 5:** Molecular docking of 1,4-Dimethyl-4-pententyl acetate, for the selected proteins.

Protein	PDB ID	5-FU	1,4-Dimethyl-4-pententyl acetate
Estrogen receptor α	3ERT	-5.4	-5.3
Estrogen receptor β	1QKM	-5.5	-5.3
HBRD4	4ZW1	-5.0	-5.1
HER 2	3PP0	-5.9	-5.8
Progesterone receptor	1E3K	-5.5	-6.0

**Figure 2:**
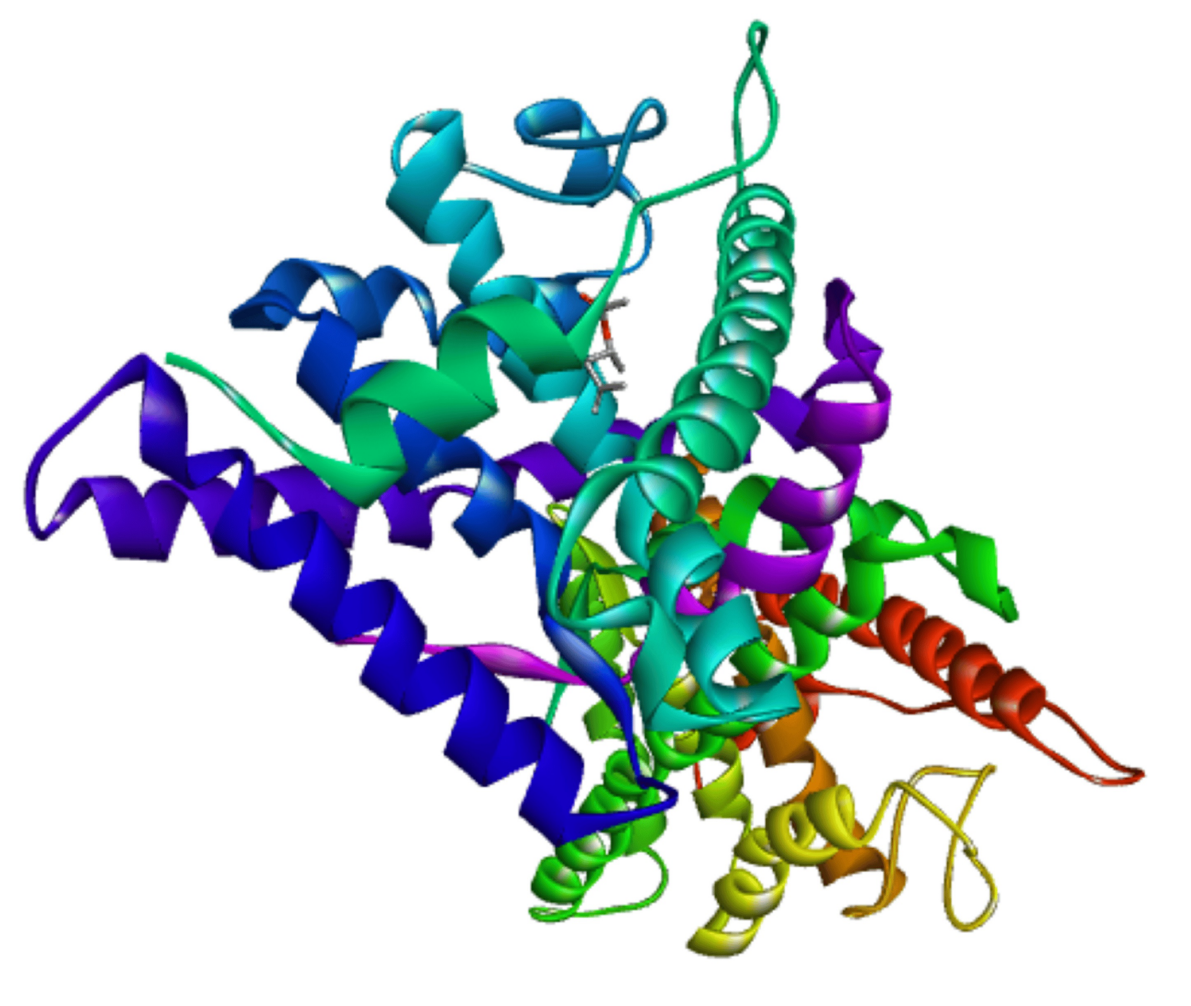
1E3K, three-dimensional structure of protein ligand complex (1,4-dimethyl-4-pententyl acetate)

**Figure 3:**
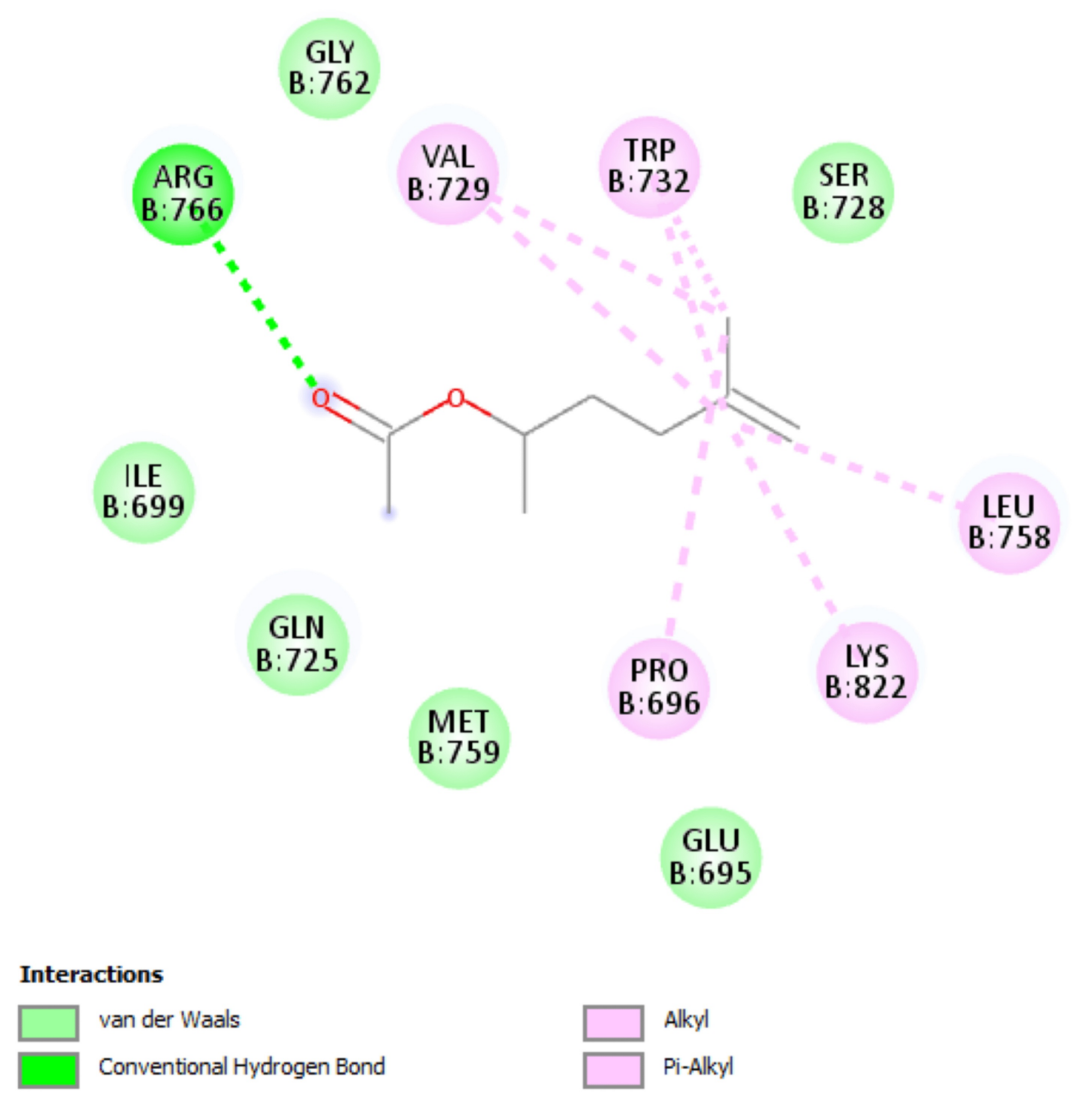
1E3K, two-dimensional interaction (1,4-dimethyl-4-pententyl acetate)

**Figure 4:**
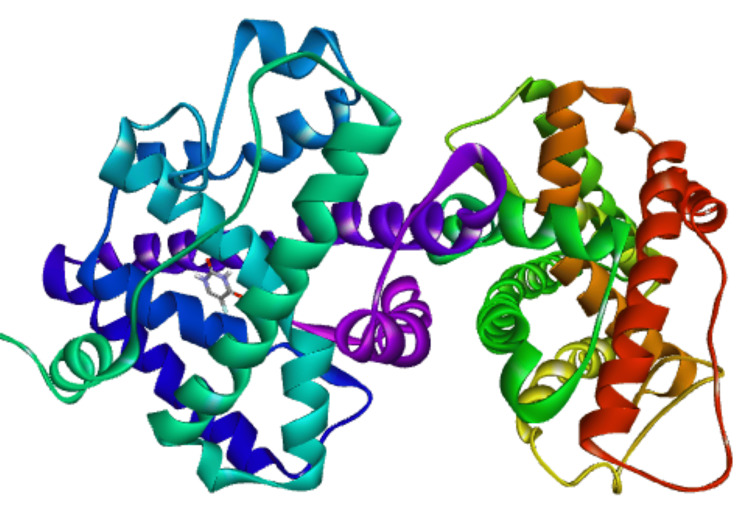
1E3K, three-dimensional structure of protein ligand complex (5-fluorouracil)

**Figure 5:**
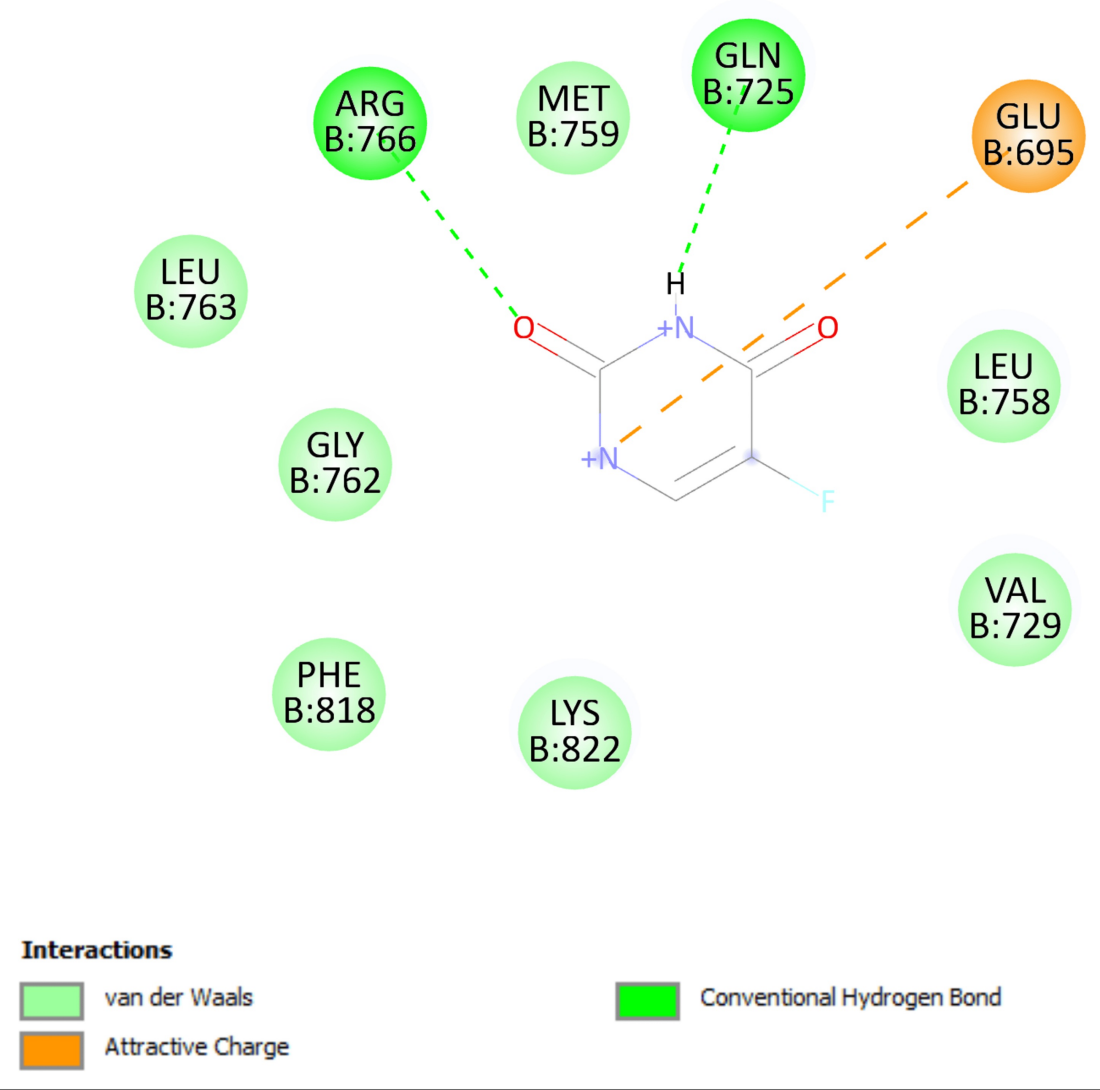
1E3K, two-dimensional interaction (5-fluorouracil)

The 3D and two-dimensional (2D) interaction of 1,4-dimethyl-4-pententyl acetate and 5-fluorouracil for the progesterone receptor (PDB ID 1E3K) are shown in Figures 2-5.

## Discussion

The ethyl acetate extract of *A. precatorius* leaves is known to have substantial efficacy towards cell lines of breast cancer [7]. Out of 97 compounds, eight compounds were selected by GC/MS analysis on the basis of compounds having a percentage area of above 2 as given in Table 1. Further molecular properties, biological activities, toxicity studies, and molecular docking were done by utilizing online computational software to identify the compound that has a better binding affinity towards the breast cancer receptor.

Molinspiration software predicted the phytochemicals of *A. precatorius*, aiding in selecting orally active compounds based on Lipinski's rule. It indicated that poor absorption or penetration was more likely when the molecular weight was larger than 500, more than 5 hydrogen bond donors, and more than 10 hydrogen bond acceptors, and the calculated Log P was not less than 5. Oral bioavailability would be affected if any violation occurs in this rule [19]. The results obtained from the Molinspiration software of predicted molecular properties are shown in Table 2.

Chemical entities of 1-octadecyne, heptacosanoic acid methyl ester, 1,1, difluoro dodecane, 1-iododecane, and phthalic acid-4-cyanophenyl nonyl ester each had one violation for miLogP. These compounds are orally inactive as these chemical entities show violation of the Lipinski rule; thus, these five compounds were removed from the study. A substance with an elevated logP score is likely to undergo rapid metabolism and due to its low water solubility, results in poor gastrointestinal absorption. A highly lipophilic chemical has a high possibility of binding to hydrophobic proteins other than the targeted target, perhaps leading to higher toxicity [12].

The bioactivity score for the selected compounds of the ethyl acetate extract of *A. precatorius* was also generated by Molinspiration. The bioactivity scores for the non-violated compounds to act as ion channel modulator, kinase inhibitor, G-protein-coupled receptor ligand, nuclear receptor ligand, enzyme inhibitor, and protease inhibitor are given in Table 3.

The bioactivity scores predicted for the selected compounds of the ethyl acetate extract of *A.* *precatorius* leaves were found to be -3.65 to 0.07, which were equivalent to the reference compounds (-3.15 to 0.30). The two compounds that were most likely to have good activity were 1,4-dimethyl-4 pentenyl acetate and 3,3-bis-tert-butylsulfanyl-2-fluoro-acrylonitrile while the other compounds showed log P below -0.5, which is the maximum extent for drug penetration across biological membranes. Therefore, these two molecules have considerable bioavailability. Bioactivity scores greater than 0.00 indicate a higher likelihood of significant biological functions, whereas scores between -0.50 and 0.00 indicate mild activity. If the score is less than -0.50, it is considered to be inactive [20]. 1,4-Dimethyl-4 pentenyl acetate also possessed moderate bioactivity for ion channel modulator (-0.40) and enzyme inhibitor (-0.22) and was inactive for GCPR, kinase inhibitor, nuclear receptor ligand, and protease inhibitor since its bioactivity score was less than -0.5. In all these cases, 3,3-dimethylhexane was inactive [21]. The findings clearly demonstrate that the physiological impacts of drug pharmacological complexes may include several mechanisms. These bioactivity scores strongly suggested a moderate interaction with all drug pharmacological targets [20].

The biological activity of the two compounds was determined for antioxidant, anti-inflammatory, immunosuppressant, antineoplastic, and cytoprotective properties by utilizing PASS software. For 3,3-bis-tert-butylsulfanyl-2-fluoro-acrylonitrile, the Pa score was less than 0.5 to the corresponding specified parameters, so it was neglected. Meanwhile, 1,4-dimethyl-4 pentenyl acetate possessed moderate bioactivity Pa scores for anti-inflammatory (0.733), immunosuppressant (0.604), and antineoplastic (0.638) (Table 4). Thus, 1,4-dimethyl-4 pentenyl acetate was further subjected to in silico toxicity studies.

The in silico toxicity of the compound (1,4-dimethyl-4 pentenyl acetate) chosen based on PASS score and molinspiration was estimated using the Osiris Property Explorer. This is an extremely important phase in drug creation since it verifies the safety of compounds. The Osiris software forecasts the potential medicinal toxicity of compounds based on structural components, and predicts the toxicity towards mutagenic, tumorigenic, and reproductive effectiveness. This in silico method surpasses animal models, which were constrained by time, ethical considerations, and fund limits. The 1,4-dimethyl-4 pentenyl acetate was proven to be safe since it showed a green signal to all the three toxicity parameters and hence, it was the least likely to be mutagenic, tumorigenic, and would do no harm to reproductive organs [12].

Molecular docking

The 1,4-dimethyl-4-pententyl acetate was docked onto the ER α, ER β, progesterone receptor, hBRD4, and HER2 to analyze the binding affinity to the proteins. The 5- fluorouracil was taken as a reference compound and showed the binding energy of -5.4 Kcal/mol on ER α, -5.5 Kcal/mol on ER β, -5.0 Kcal/mol on hBRD4, -5.5 Kcal/mol on progesterone receptor, and -5.9 Kcal/mol on HER 2 receptors, while 1,4-dimethyl-4-pententyl acetate showed the binding energy of -5.0 Kcal/mol on ER α, -5.3 Kcal/mol on ER β, -5.1 Kcal/mol on hBRD4, -6.0 Kcal/mol on progesterone receptor, and -5.8 Kcal/mol on HER 2 receptors. Binding affinities are given in Table 5. A better binding affinity was shown by 1,4-dimethyl-4-pententyl acetate towards progesterone and HER 2 receptors. Of this, the binding affinity towards the progesterone receptor was greater when compared to the reference compound 5-fluorouracil.

ER α is a key therapeutic target for breast cancer. The human estrogen hormone plays a vital role in the development of breast carcinoma. ERs are unique hormone receptors that appear to be highly expressed in two-thirds of cancer cells connected with breast cells [11].

As a possible epigenetic therapeutic target, BRD4 inhibitors were presently undergoing preclinical and clinical trials for the treatment of various tumor types. BRD4 can be specifically inhibited to suppress growth and trigger apoptosis in a variety of cancerous cells, such as diffuse large B-cell lymphoma, breast and prostate cancer, and acute myeloid leukemia. BRD4 regulates the malignancy of breast cancer cells and is upregulated in breast cancer cells [22].

HER2 is a tyrosine kinase protein [14] and it is the subpart of the HER/estimated glomerular filtration rate (EGFR)/erythroblastic leukemia viral oncogene homolog (ERBB) family, which also includes HER1, HER3, and HER4. There was a unique family of oncogenic proteins whose amplification has been demonstrated to play a vital role in the genesis as well as the progression of a certain aggressive type of breast cancer [23].

ER β belongs to the superfamily of nuclear transcription factors. In breast cancer, ER β functions as a separate prognostic and predictive factor [24].

The progesterone receptor, also known as a component of the nuclear receptor (NR) superfamily, controls a wide range of genes. Ligand interaction with progesterone produces a conformational alteration in the receptor, allowing progesterone to bind to genetic material DNA and influence transcription. Lack of progesterone expression is linked to a poor overall prognosis and survival in breast cancer patients [25].

The 3D interaction of 1,4-dimethyl-4-pententyl acetate and 5 fluorouracil with progesterone receptors is shown in Figure 2 and Figure 4, respectively. The 2D interaction of 1,4-dimethyl-4-pententyl acetate with progesterone receptor was noted to form one conventional hydrogen bond with ARG B:766, two π alkyl bond with TRP B:732 and one alkyl bond with each of LEU B:758, LYS B:822, PRO B:696, and two alkyl bond with VAL B:729, shown in Figure 3. Whereas the interaction of 5-fluorouracil with progesterone receptor was noted to form two conventional hydrogen bonds with ARG B:766 & GLN B:725, one attractive charge bond with GLU B:695, shown in Figure 5.

Limitation

This study has some limitations. In this study, only in silico studies were done and the in silico models provide only predicted characteristics for the compounds being analyzed. Further, the compound 1,4-dimethyl-4-pententyl acetate is moderately active since the bioactivity score is between -0.50 and 0.00. In future studies, some structural modifications will be made to make the compound active, and also some cell line studies will be performed to confirm the activity toward breast cancer.

## Conclusions

The utilization of natural substances has the potential to be both medicinal and nutritive for people. Numerous studies have demonstrated that biological products are a better option than synthetic and chemical pharmaceuticals because they have no adverse effects and are naturally derived. In recent decades, quantitative structural-activity relationship (QSAR)-based computational techniques have been used to assess drug physicochemical parameters, drug probability, and toxicity. In this investigation, the lead drug was found using computational approaches with a possible effect on the selected breast cancer receptors. The findings suggest that the physiological activities of the drug complexes may be mediated by many routes, including interactions with GPCR ligands, kinase inhibitors, nuclear receptor ligands as well as protease and enzyme inhibitors. The compound is non-mutagenic, nontumorigenic, and has no reproductive effectiveness with high drug score values. The in silico estimation of oral bioavailability (Lipinski rule of five) and the ADMET (absorption-distribution-metabolism-excretion-toxicity) risk analysis are within acceptable ranges for active entities. 

The lead chemical selected for molecular docking research was 1,4-dimethyl-4-pententyl acetate. This chemical binds to ERs α, β, progesterone, hBRD4, and HER2 receptors. When compared to the reference chemical, 1,4-dimethyl-4-pententyl acetate had a higher affinity for the progesterone receptor when compared to the reference compound (5-fluorouracil). In hormone-dependent malignancies such as endometrial and breast cancer, progesterone receptors play a critical role by stimulating cell division and preventing apoptosis. The discovery of lead compounds that target progesterone receptors is an emerging area of research in cancer therapy. Lead compounds are possible therapeutic agents that have demonstrated effectiveness in altering progesterone receptor activity to achieve anticancer activity. The lead chemicals that target progesterone receptors can affect the course of cancer via different mechanisms. Further investigation into these substances may result in less harmful and more effective cancer treatments, especially for those with hormone-dependent malignancies. Based on the biological activity scores, 1,4-dimethyl-4-pententyl acetate is somewhat active; therefore, in the future, certain modifications need to be done to make the compound active, and further in vitroand in vivo studies must be conducted to validate the research.

## References

[1] Tzenios N, Tazanios ME, Chahine M (2024). The impact of BMI on breast cancer - an updated systematic review and meta-analysis. Medicine (Baltimore).

[2] Manoochehri H, Farrokhnia M, Sheykhhasan M, Mahaki H, Tanzadehpanah H (2024). Key target genes related to anti-breast cancer activity of ATRA: a network pharmacology, molecular docking and experimental investigation. Heliyon.

[3] Yap KM, Sekar M, Seow LJ (2021). Mangifera indica (mango): a promising medicinal plant for breast cancer therapy and understanding its potential mechanisms of action. Breast Cancer (Dove Med Press).

[4] Chhikara BS, Parang K (2023). Global cancer statistics 2022: the trends projection analysis. Chem Biol Lett.

[5] Aswin RK, Tridiganita IS, Arif NM (2022). Abrus precatorius: a comprehensive insight into the phytochemical, pharmacological, therapeutic activities and safety. J Drug Delivery Therapeut.

[6] Solanki A, Zaveri M (2012). Pharmacognosy, phytochemistry and pharmacology of Abrus precatorius leaf: a review. Int J Pharma Sci Rev Res.

[7] Sofi MS, Sateesh MK, Bashir M, Ganie MA, Nabi S (2018). Chemopreventive and anti-breast cancer activity of compounds isolated from leaves of Abrus precatorius L. 3 Biotech.

[8] Gul MZ, Ahmad F, Kondapi AK, Qureshi IA, Ghazi IA (2013). Antioxidant and antiproliferative activities of Abrus precatorius leaf extracts--an in vitro study. BMC Complement Altern Med.

[9] Kaula BC, Mishra R, Geeta Geeta (2022). Phytoconstituents and ethnopharmacological activities of Abrus precatorius L. (Fabaceae): a review. Vegetos.

[10] Bhattacharjee A, Hossain MU, Chowdhury ZM, Rahman SM, Bhuyan ZA, Salimullah M, Keya CA (2021). Insight of druggable cannabinoids against estrogen receptor β in breast cancer. J Biomol Struct Dyn.

[11] Muhammad S, Saba A, Khera RA (2022). Virtual screening of potential inhibitor against breast cancer-causing estrogen receptor alpha (ERα): molecular docking and dynamic simulations. Mol Simul.

[12] Hachani K, Othmani F, Essam M, Akhtar MJ, Khan S (2023). In silico studies on olive oil polyphenolic natural products to identify neuroprotective lead compounds beneficial in the treatment of Alzheimer's disease. Arab J Med Aromatic Plant.

[13] Acharya R, Chacko S, Bose P, Lapenna A, Pattanayak SP (2019). Structure based multitargeted molecular docking analysis of selected Furanocoumarins against breast cancer. Sci Rep.

[14] Dhiwar PS, Matada GS, Raghavendra NM (2022). Current updates on EGFR and HER2 tyrosine kinase inhibitors for the breast cancer. Med Chem Res.

[15] Dhorajiwala TM, Halder ST, Samant L (2019). Comparative in silico molecular docking analysis of l-threonine-3-dehydrogenase, a protein target against African trypanosomiasis using selected phytochemicals. J App Biotech Rep.

[16] Jha V, Devkar S, Gharat K (2022). Screening of phytochemicals as potential inhibitors of breast cancer using structure based multitargeted molecular docking analysis. Phytomed Plus.

[17] Yahaya MA, Bakar AR, Stanslas J, Nordin N, Zainol M, Mehat MZ (2021). Insights from molecular docking and molecular dynamics on the potential of vitexin as an antagonist candidate against lipopolysaccharide (LPS) for microglial activation in neuroinflammation. BMC Biotechnol.

[18] Begum RF, Mohan S (2024). Insights into vitamin E with combined oral contraceptive on INSR gene in PCOS by integrating in silico and in vivo approaches. Appl Biochem Biotechnol.

[19] Lipinski CA, Lombardo F, Dominy BW (2012). Experimental and computational approaches to estimate solubility and permeability in drug discovery and development settings. Adv Drug Deliv Rev.

[20] Srivastava R (2021). Theoretical studies on the molecular properties, toxicity, and biological efficacy of 21 new chemical entities. ACS Omega.

[21] Suganya M, Hemamalini M, JoseKavitha S, Rajakannan V (2020). Insilico studies of molecular property and bioactivity of organic crystalline compounds using Molinspiration. Int Res J Eng Tech.

[22] Lu L, Chen Z, Lin X (2020). Inhibition of BRD4 suppresses the malignancy of breast cancer cells via regulation of Snail. Cell Death Differ.

[23] Arannilewa AJ, Suleiman Alakanse O, Adesola AO (2018). Molecular docking analysis of Cianidanol from Ginkgo biloba with HER2+ breast cancer target. Bioinformation.

[24] Zhou Y, Liu X (2020). The role of estrogen receptor beta in breast cancer. Biomark Res.

[25] Zarezade V, Abolghasemi M, Rahim F, Veisi A, Behbahani M (2018). In silico assessment of new progesterone receptor inhibitors using molecular dynamics: a new insight into breast cancer treatment. J Mol Model.

